# Pesticide-mediated interspecific competition between local and invasive thrips pests

**DOI:** 10.1038/srep40512

**Published:** 2017-01-13

**Authors:** Xueyin Zhao, Stuart R. Reitz, Huiguo Yuan, Zhongren Lei, Dean Ronald Paini, Yulin Gao

**Affiliations:** 1 State Key Laboratory for Biology of Plant Diseases and Insect Pests, Institute of Plant Protection, Chinese Academy of Agricultural Sciences, Beijing 100193, China; 2 Malheur County Extension, Department of Crop and Soil Science, Oregon State University, 710 SW 5th Ave, Ontario, OR 97914, USA; 3 CSIRO, Black Mountain Laboratories, Acton, ACT 2601, Australia

## Abstract

Competitive interactions between species can be mitigated or even reversed in the presence of anthropogenic influences. The thrips species *Frankliniella occidentalis* and *Thrips tabaci* are highly invasive and damaging agricultural pests throughout the world. Where the species co-occur, one species tends to eventually predominate over the other. Avermectin and beta-cypermethrin are commonly used insecticides to manage thrips in China, and laboratory bioassays demonstrated that *F. occidentalis* is significantly less susceptible than *T. tabaci* to these insecticides. In laboratory cage trials in which both species were exposed to insecticide treated cabbage plants, *F. occidentalis* became the predominant species. In contrast, *T. tabaci* completely displaced *F. occidentalis* on plants that were not treated with insecticides. In field trials, the species co-existed on cabbage before insecticide treatments began, but with *T. tabaci* being the predominant species. Following application of avermectin or beta-cypermethrin, *F. occidentalis* became the predominant species, while in plots not treated with insecticides, *T. tabaci* remained the predominant species. These results indicate that *T. tabaci* is an intrinsically superior competitor to *F. occidentalis,* but its competitive advantage can be counteracted through differential susceptibilities of the species to insecticides. These results further demonstrate the importance of external factors, such as insecticide applications, in mediating the outcome of interspecific interactions and produce rapid unanticipated shifts in the demographics of pest complexes.

High competitive ability is believed to be an important characteristic of invasive species^1^. Many researchers have demonstrated the competitive superiority of an invading species by comparing its competitive abilities with those of a native species that is being displaced^2^,^3^,^4^,^5^. The coexistence of an invasive species and native species is rarely stable, with one species typically displacing the other within a relatively short period of time^1^. Although interspecific competition is major factor in displacements, other ecological mechanisms contribute to displacements.

*Frankliniella occidentalis* (Pergande) is native to western North America but in the last 30 years, has become a worldwide invasive pest^6^,^7^. This species has become the dominant phytophagous thrips in many of the regions it has invaded, including Japan^8^, Spain^9^, Turkey^10^ and Argentina^11^. In European greenhouses, *F. occidentalis* has replaced *Thrips tabaci* Lindeman as the major thrips pest. Van Rijn, Mollena & Steehuis-Broers^12^ suggest that *F. occidentalis* is a superior competitor, after finding no difference between the two species in either the intrinsic rate of increase, net reproductive time, or development times. More recently, Northfield *et al*.^13^ showed that *F. occidentalis* is competitively superior to *F. bispinosa* (Morgan), a common thrips species of southern Florida, USA. In contrast, Paini *et al*.^14^ showed that the invasive *F. occidentalis* appears to be competitively excluded by the native *F. tritici* (Fitch), and they suggest that the likely mechanism of competition between the two species was interference rather than exploitative competition. In China, *F. occidentalis* was first found in Beijing in 2003, and has since spread throughout China^15^. By 2014, *F. occidentalis* was found in more than 10 provinces throughout China^16^, where it has rapidly become a significant horticultural pest.

*Thrips tabaci* is native to southern Asia, and can occur at high densities resulting in significant damage to crops^15^. Currently, this species is still an important pest thrips throughout vegetable growing regions of China. Although *T. tabaci* and *F. occidentalis* share many of the same host crops, and co-exist in some crops and regions, interspecific interactions between them remain unclear. *F. occidentalis* is largely absent or occurs in low numbers in some regions, where the native *T. tabaci* predominates^15^. In contrast, *T. tabaci* is largely absent or occurs in low numbers in some regions where the invasive *F. occidentalis* has become predominant^15^. It is possible that external factors determine which species competitively excludes the other from a particular region.

One factor that can mediate competitive interactions is the use of insecticides in cropping systems. Research has demonstrated that differential susceptibility to insecticides may increase the likelihood of one species being replaced by another, thus driving changes in population demographics^17^,^18^,^19^. Two of the most commonly applied insecticides for management of thrips in China are avermectin and beta-cypermethrin^15^. Using both laboratory and field experiments, we investigated interspecific differences in susceptibility to these insecticides and determined if differential susceptibility could explain the observed changes in thrips demographics.

## Results

### Baseline toxicity bioassays

Probit analyses showed that the respective lethal concentrations of both insecticides were significantly higher for *F. occidentalis* than for *T. tabaci* (Table 1). Control mortality was <5% in all replicates. Avermectin was significantly more toxic than beta-cypermethrin to *T. tabaci.* However based on the 95% confidence limits, the respective LC_90_ values were not significantly different from the recommended field rates of 1 ppm for avermectin and 5 ppm for beta-cypermethrin. Based on the susceptibility ratios, the differences between the species were greater for avermectin than for beta-cypermethrin. *F. occidentalis* was approximately 10 times less susceptible than *T. tabaci* to beta-cypermethrin (Table 1). The difference in susceptibility between the species to beta-cypermethrin was four fold greater than that for avermectin.

### Thrips response to insecticides in laboratory experiments

**Figure 1:**
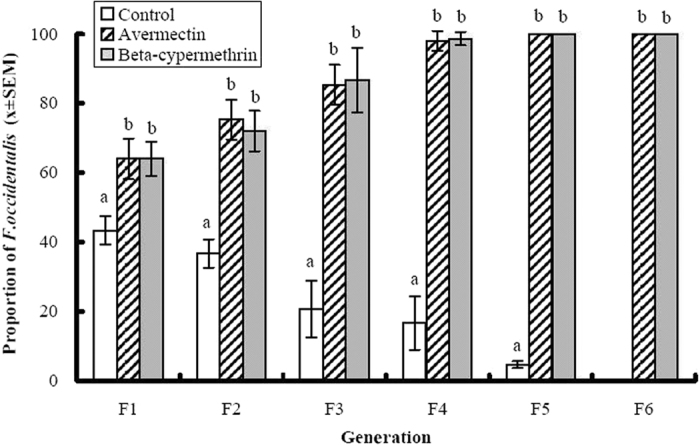
The effects of avermectin (1 ppm) and beta-cypermethrin (5 ppm) on the proportion of *Frankliniella occidentalis* among adult thrips collected from purple cabbage plants infested with both *F. occidentalis* and *Thrips tabaci* in the cage experiments. Columns represent mean proportions and error bars represent standard error of the means. Means within the same date marked by different lower case letters are significantly different at P = 0.05 level according to least squares means procedures. Values on the *x-*axis denote generations of thrips following initial release of insects into cages. Insecticides were applied after the initial thrips release and following insect collections after the F1 and F2 generations.

There was a significant interaction between insecticide treatments and time on the proportions of *F. occidentalis* (F = 9.88, df = 10, 30, P < 0.001). The proportions of *F. occidentalis* in the avermectin and beta-cypermethrin treatments were significantly greater than in the control treatment (F = 54.24, df = 2,6, P < 0.0001). This pattern was evident on each of the sample dates after the insecticide applications began (Fig. 1). Not only were the proportions of *F. occidentalis* in the insecticide treatments greater than those in the control treatment, the magnitude of the differences increased over time, which accounted for the significant insecticide treatment by time interaction. Despite being introduced at the initial population ratio of 1:1, there were significantly more *T. tabaci* in the control treatment by the second generation. *T. tabaci* completely displaced *F. occidentalis* by the sixth generation in the control treatment. However, this pattern was reversed when plants were treated with either avermectin or beta-cypermethrin. In those treatments, *F. occidentalis* became the dominant species and completely displaced *T. tabaci* after the fifth generation (Fig. 1.). However, there were no significant differences in the proportions of *F. occidentalis* between the avermectin and beta-cypermethrin treatments on any of the sample dates (Fig. 1).

### Thrips response to insecticides in field experiments

**Figure 2:**
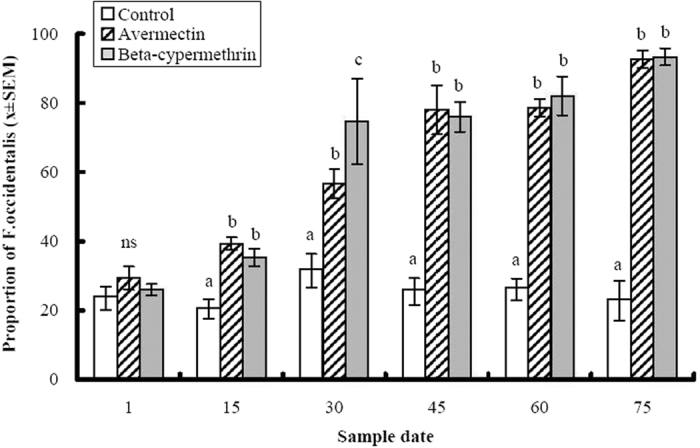
The effects of avermectin (1 ppm) and beta-cypermethrin (5 ppm) on the proportion of *Frankliniella occidentalis* among thrips adults collected over time on purple cabbage plants grown in Wucheng County where both *F. occidentalis* and *T. tabaci* occur. Columns represent mean proportions and error bars represent standard error of the means. Means within the same date marked by different lower case letters are significantly different at P = 0.05 level according to least squares means procedures. Sample dates listed on the *x*-axis denotes days after the initiation of the experiment. Insecticides were applied after the collection of thrips samples on days 1, 15 and 30 of the experiment.

**Figure 3:**
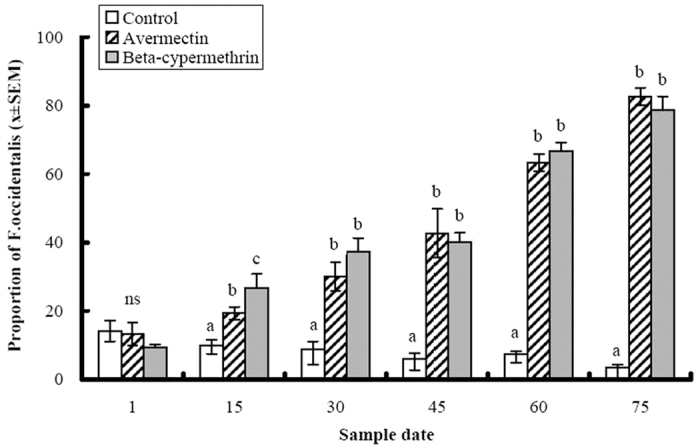
The effects of avermectin (1 ppm) and beta-cypermethrin (5 ppm) on the proportion of *Frankliniella occidentalis* among thrips adults collected over time on purple cabbage plants grown in Xiajin County where both *F. occidentalis* and *T. tabaci* occur. Columns represent mean proportions and error bars represent standard error of the means. Means within the same date marked by different lower case letters are significantly different at P = 0.05 level according to least squares means procedures. Sample dates listed on the *x*-axis denotes days after the initiation of the experiment. Insecticides were applied after the collection of thrips samples on days 1, 15 and 30 of the experiment.

The only thrips species collected in the field experiments were *F. occidentalis* and *T. tabaci*. Initially, *T. tabaci* was the predominant species at both locations, comprising 74 and 88% of adult thrips at Wucheng and Xiajin, respectively before the insecticide applications began (Figs 2 and 3). At Wucheng County, there was a significant interaction between insecticide treatments and time (F = 12.75, df = 10, 30, P < 0.0001). Although the proportions of *F. occidentalis* adults collected did not differ among the three treatments on the first sample date, which was before any insecticides were applied, the insecticide treatments significantly altered the relative abundance of *F. occidentalis* over the course of the experiment (F = 109.18, df = 2, 6, P < 0.0001). In the control treatment, the relative proportions of the species remained consistent over time and *T. tabaci* remained the predominant species (Fig. 2). However, there were increasingly greater proportions of *F. occidentalis* in the avermectin and beta-cypermethrin treatments on days 15, 30, 45, 60 and 75 of the experiment (Fig. 2). By the end of the trial, the percentages of *F. occidentalis* in the avermectin and beta-cypermethrin treatments were approximately 93%, whereas it remained approximately 23% in the control treatment.

Similar patterns were evident in the field trial at Xiajin County. There was a significant interaction between insecticide treatment and time (F = 27.09, df = 10, 30, P < 0.0001). The composition of the thrips complex changed over time (F = 45.70, df = 10, 30, P < 0.0001), but the changes were consistent with time. *T. tabaci* was the predominant species at the beginning of the trial, comprising 88% of the thrips collected before insecticide applications began. *T. tabaci* remained the predominant species in the control treatment over the duration of the experiment, where its relative abundance increased from 86% to 97% at the conclusion of the trial (Fig. 3). An opposite trend occurred in the avermectin and beta-cypermethrin treatments. The relative abundance of *F. occidentalis* was significantly greater in the two insecticide treatments than in the control treatment (F = 155.74, df = 2, 6, P < 0.0001). The proportions of *F. occidentalis* adults differed between the insecticide treatments and the control treatment following the first insecticide application, and this difference increased over time, with the proportion of *F. occidentalis* exceeding 80% by the end of the trial. These results indicate that applications of avermectin and beta-cypermethrin reduced the abundance of *T. tabaci* relative to the abundance of *F. occidentalis* (Fig. 3).

## Discussion

Our results demonstrate that *T. tabaci* is competitively superior to *F. occidentalis* on cabbage foliage in the absence of insecticides. However, the field and laboratory populations of *F. occidentalis* in our trials were significantly less susceptible to avermectin and beta-cypermethrin than were sympatric populations of *T. tabaci.* These differences in insecticide susceptibility could be driving the displacement of *T. tabaci* by *F. occidentalis* in agricultural settings, although *T. tabaci* currently is still the predominant thrips of horticultural crops in China, and avermectin and beta-cypermethrin have been used for thrips management for many years throughout China. These two insecticides constitute more than 40–60% of the insecticide use against thrips on horticultural crop growing regions, with avermectin being the predominant insecticide. The thrips populations that we used in our laboratory trials were collected from the same fields in Shandong Province. Consequently, they would have experienced similar insecticide exposures. Further, the field trials were conducted in areas where *F. occidentalis* and *T. tabaci* still co-occur, and thus those populations would have had similar histories of insecticide exposure.

Our laboratory cage experiments demonstrated that, in the absence of insecticides treatments, *T. tabaci* outcompeted *F. occidentalis* and completely displaced the invasive *F. occidentalis* in a very short time period. Cabbage has been shown to be a highly suitable host for both species when examined in single species trials^20^,^21^,^22^. However in mixed species trials, the reproductive capacity and ratio of females to males of *F. occidentalis* decreases significantly in the presence of *T. tabaci*^22^. Thus, the possible mechanisms responsible for competitive displacement of *F. occidentalis* by *T. tabaci* may include differential fecundity between the two species and differential larval survivorship on cabbage foliage.

The results of the laboratory cage show how the demographics of thrips populations can change rapidly in response to insecticide applications. Samples collected before the application of any insecticide indicated that the two species were present in near equal proportions for the three treatments. However, immediately after insecticide applications began, the proportion of *F. occidentalis* in the insecticide treatments increased. These changes were likely driven by the greater susceptibility of *T. tabaci* to avermectin and beta-cypermethrin. The field experiment further indicated that *F. occidentalis* was significantly more tolerant to avermectin and beta-cypermethrin than was *T. tabaci,* and such differences in susceptibility could be driving the displacement of *T. tabaci* by *F. occidentalis. Frankliniella occidentalis* tends to proliferate in vegetable crops in Florida, USA when broad-spectrum insecticides eliminate more susceptible competitor thrips species and predator species^23^. In that region, *F. occidentalis* becomes exceedingly rare on non-crop hosts outside of agricultural fields^24^,^25^.

Differential responses of insect populations to insecticides may not be limited to the toxic effects of the insecticides^26^,^27^. Our baseline toxicity bioassays showed that the LC_90_ values for *T. tabaci* were similar to the recommended field rates for avermectin and beta-cypermethrin, whereas the values were significantly higher for *F. occidentalis,* which suggests that *F. occidentalis* may have been subject to sublethal effects of the insecticides. Sublethal concentrations of insecticides may still have deleterious effects on survivors, including prolonging development times or reducing subsequent adult fecundity, as has been demonstrated for avermectin and beta-cypermethrin^28^,^29^. However, sublethal concentrations of insecticides may also induce positive effects on insect bionomics^26^,^27^. Sublethal concentrations of beta-cypermethrin lead to increased fecundity of *Harmonia axyridis*^30^. When treated with sublethal concentrations of avermectin as immatures, *Panonychus citri* have shorter generation doubling times and greater intrinsic growth rates than untreated immatures^28^.

However, the phenomenon of *T. tabaci* completely displacing *F. occidentalis* that occurred in the control treatment of our cage experiment was not observed in the field experiments. Although *T. tabaci* was a superior competitor to the invasive *F. occidentalis* on purple cabbage plants, competitive asymmetry alone may not result in the complete exclusion of a species on a single host. *Frankliniella occidentalis* may have colonized the untreated cabbage from other sources, including from the insecticide treated cabbage. Therefore, it is not surprising that *F. occidentalis* was still present in the control treatments in this field community, though at very low densities. Likewise, *T. tabaci* was not completely excluded in the insecticide treatment plots. This result may also reflect dispersal of *T. tabaci* into plots from outside sources, including the untreated cabbage. It also is possible that other biological mechanisms or anthropogenic factors operating in the open field conditions could influence competitive interactions between these species.

Differential susceptibility to insecticides has been linked to changes in other pest complexes. For example, in the USA, the spirea aphid, *Aphis spireacola* Patch has displaced *A. pomi* De Geer in apple (*Malus domestica* Borkh)^31^,^32^,^33^. These studies showed that *A. spireacola* is significantly less susceptible than *A. pomi* to a range of commonly used aphicides. Further, *A. pomi* is able to persist at low levels in the apple system because it is less likely to disperse from apple than is *A. spireacola*^33^. Exposure to sublethal doses of nitenpyram increases the susceptibility of *Trialeurodes vaporariorum* to other insecticides but it does not affect the susceptibility of *Bemisia tabaci* (Gennadius). This differential susceptibility to insecticides may have allowed *B. tabaci* to become the predominant whitefly species in northern China^19^. Similarly, the B biotype of *B. tabaci* has been displaced by the Q biotype in many regions has been attributed to the greater insecticide resistance of the Q biotype^34^,^35^. This displacement has occurred despite the fact that B biotype is a superior competitor^36^. Furthermore, the displacement of *Liriomyza sativae* Blanchard by *Liriomyza trifolii* (Burgess) in China and USA where has been attributed to the greater insecticide tolerance of *L. trifolii*^17^.

Given that we found *T. tabaci* were more sensitive than *F. occidentalis* to avemectin and beta-cypermethrin, changes in population demographics following insecticide applications would be expected to occur. These findings indicate the importance of accurate species identification in pest management programs. Continued use of either avermectin or beta-cypermethrin at the recommended rates could lead to apparent control failures in the field, especially in cases where *F. occidentalis* displaces *T. tabaci* because of differential insecticide susceptibility and become the predominant species in a growing region. The results of the current experiments indicate that differential susceptibility to insecticides may trigger the competitive exclusion of *T. tabaci,* with continued insecticide use accelerating the rate of displacement. Development of resistance to either of these two major insecticides used in China, would be problematic because of the lack of alternatives^37^. To help mitigate problems with either species, integrated pest management (IPM) programs that emphasize the conservation of biological control agents or use of microbial biopesticides should be implemented^23^,^37^,^38^,^39^,^40^,^41^,^42^,^43^,^44^.

The results reported here provide evidence that *T. tabaci* is competitively superior to the invasive *F. occidentalis,* and in areas where *T. tabaci* currently predominates, it may be deter the establishment of large populations of *F. occidentalis*. However, the results reported here also provide evidence that the direction of species displacement between these thrips species can change due to their differential susceptibility to commonly used insecticides.

## Methods

### Insect strains and rearing

Populations of both *F. occidentalis* and *T. tabaci* were collected from purple cabbage, *Brassica oleracea* L. var. capitata, in Dezhou, Shandong Province, in June 2013. The field where thrips were collected was not treated with insecticides, but surrounding fields in the area did receive insecticide treatments. *F. occidentalis* and *T. tabaci* were reared separately on purple cabbage plants in the absence of insecticides at the Dezhou Agricultural Experiment Station under controlled conditions (25 ± 2 °C, 60 ± 10% RH, 16:8 h L:D). Thrips were reared for one generation before being used in experiments. *T. tabaci* were an all female thelytokous population. *F. occidentalis* were arrhenotokous, with a female-biased sex ratio (~75% female). Adult females of *F. occidentalis* are typically larger than those of *T. tabaci* although there is overlap in their sizes^45^.

### Baseline toxicity bioassays

The bioassay technique used to determine differential susceptibility of *F. occidentalis* and *T. tabaci* to avermectin and beta-cypermethrin was similar to the adult bioassay methods described by Wang *et al*.^46^. Only recently eclosed (1 day post eclosion) adult females were used in bioassays. Each insecticide was serially diluted to 6–7 concentrations with distilled water containing 0.1% Triton X-100 (Beijing Solar Bio Science and Technology Co. Ltd., China). Commercial formulations of avermectin and beta-cypermethrin insecticides (Hebei Veyong Bio-Chemical) were used in all trials. Leaves were cut from purple cabbage plants grown in a greenhouse without any insecticides, and then were dipped in the appropriate insecticide concentration for 10 s. Control leaves were treated in the same manner with a 0.1% Triton X-100 solution. Leaves were allowed to dry at room temperature for approximately 1 hour.

Modified ventilated glass cells were used as the bioassay chambers. A glass cell consisted of a center glass plate (4 mm thick) with two holes (20 mm diameter) drilled through the plate. The center glass plate was covered by a solid top and a bottom glass plate. The three plates were kept together with strong rubber bands forming two cells. Two ventilation holes were drilled opposite each other in the sides of the plate into the cell. The holes were covered inside the cell by copper wire screen with a mesh size of 200 μm.

After air-drying, each leaf was placed with its adaxial surface downward onto filter paper. The leaf and filter paper were placed individually between the center and bottom glass of a plate. Thrips were then introduced into the cells with the experimental leaves. Thirty adult female thrips were moved into each cell with an aspirator. Three cells were prepared for each concentration (total 90 adult thrips for each concentration) and kept under the environmental conditions described above. Mortality was recorded 48 h later. Thrips unable to move and showing characteristic effects of insecticide intoxication were scored as dead.

The data from these insecticide toxicity assays were subjected to probit analysis using POLO-PC (LeOra Software, Berkeley, CA) after correcting for control mortality with Abbott’s formula^47^. Lethal concentration values (LC) were compared based on their 95% confidence limits, with non-overlapping intervals used to establish significance. Relative susceptibility ratios for the two species were calculated by dividing the LC estimates of *F. occidentalis* by the corresponding LC estimates for *T. tabaci.*

### Thrips response to insecticides in laboratory experiments

Maximum field rates of avermectin (1 ppm) and beta-cypermethrin (5 ppm) were used to determine the response of each species to these insecticides when both species were present on the same plant. Rates are based on the insecticide registration labels. Five hundred recently eclosed adults of each *F. occidentalis* and *T. tabaci* were added to a cage (100 × 100 × 100 cm) containing 15 purple cabbage plants (15–18 days old). After adults dispersed inside the cage, plants were sprayed until runoff with one of three insecticide treatments: avermectin (1 ppm), beta-cypermethrin (5 ppm) or a control (distilled water). Sampling for adult thrips was conducted on the F1, F2, F3, F4, F5 and F6 generations of the experiments. Generations were based on the eclosion of adult progeny, with generation times for these trials lasting approximately 15 days. Insecticide treatments were applied again after thrips samples were collected from the F1 and F2 generations. No additional insecticide applications were made after the F2 generation. To determine the relative species composition, fifty adult thrips were randomly collected from within each cage on each sampling date, and they were identified to species, under a dissecting microscope. All treatments were replicated three times.

The proportions of *F. occidentalis* among the mixed species in each generation were then compared using generalized linear models, incorporating a binomial distribution of the response data and a logit link. Treatment means were separated using the least squares means option^48^.

### Thrips response to insecticides in field experiments

Field experiments were conducted in Wucheng and Xiajin Counties, located in Dezhou City, Shandong Province in 2015, where *F. occidentalis* and *T. tabaci* co-occur (Gao *et al*., unpublished data). Purple cabbage was planted in both counties on 1 April at sites where *F. occidentalis* and *T. tabaci* were previously observed to co-occur. A randomized complete block experimental design was utilized, with three replications of each of three treatments. The three treatments were avermectin applied at 1 ppm, beta-cypermethrin applied at 5 ppm, and distilled water. The hand-pump type sprayer used to apply treatments was calibrated to deliver 1300 L/ha at 20–30 kPa, with 90 μm openings in the nozzles. A 5 m space without any crops was set up between plots to minimize potential insecticide contamination among plots. Each plot was approximately 66.7 m^2^ and seeded at a rate expected to produce 200 plants per plot (30,000 per hectare), as is common in this farming system. Purple cabbage was maintained using the standard agronomic practices for each of the local areas, except no other insecticides were used throughout the season.

Sampling began on 10 May and was conducted on days 1, 15, 30, 45, 60 and 75 of the experiment. Insecticides were applied after the collection of thrips samples on days 1, 15 and 30 of the experiment; no insecticide was applied on day 45, 60 and 75. Five sites were randomly chosen for sampling within each plot on each sampling date. Ten adult thrips were collected from each of the sampling sites for a total of 50 adults collected per plot per date. Thrips were identified to species under a dissecting microscope.

For these field experiments, the proportions of *F. occidentalis* on each sample date were compared by utilizing generalized linear models, incorporating a binomial distribution of the response data and a logit link. Data were analyzed separately for the trials conducted at Wucheng and Xiajin. Treatment means were separated using the least squares means option^45^.

## Additional Information

**How to cite this article**: Zhao, X. *et al*. Pesticide-mediated interspecific competition between local and invasive thrips pests. *Sci. Rep.* **7**, 40512; doi: 10.1038/srep40512 (2017).

**Publisher's note:** Springer Nature remains neutral with regard to jurisdictional claims in published maps and institutional affiliations.

## Figures and Tables

**Table 1:** Susceptibility of *Frankliniella occidentalis* and *Thrips tabaci* to avermectin and beta-cypermethrin in leaf dip bioassays.

Species	*n*	χ^*2*^	df	*P*	Slope + SE	LC_50_ (ppm)	95% CI	SR_50_^a^	LC_90_ (ppm)	95% CI	SR_90_^a^
Avermectin											
* F. occidentalis*	720	3.64	5	0.602	1.70 + 0.13	5.943	4.969–7.045	—	33.625	22.018–46.625	—
* T. tabaci*	720	3.47	5	0.628	1.68 + 0.12	0.149	0.126–0.175	39.9	0.864	0.676–1.174	38.9
Beta- cypermethrin											
* F. occidentalis*	630	9.86	4	0.043	1.65 + 0.14	8.133	4.892–11.978	—	48.43	28.91–130.03	—
* T. tabaci*	630	3.47	4	0.482	1.68 + 0.14	0.829	0.678–0.989	9.8	4.795	3.733–6.656	10.1

^a^SR – Susceptibility ratio = LC_i_ (*F. occidentalis)/*LC_i_ (*T. tabaci*).
